# Venlafaxine-induced spontaneous ejaculation: Case report and literature review

**DOI:** 10.1192/j.eurpsy.2022.877

**Published:** 2022-09-01

**Authors:** I. Gundogmus, S. Tekin, A.B. Tasci, Ö. Uzun

**Affiliations:** 1 Kirikkale Yuksek Ihtisas Hospital, Psychiatry, Kırıkkale, Turkey; 2 Gulhane Research and Training Hospital, Psychiatry, Ankara, Turkey

**Keywords:** Venlafaxine, spontaneous ejaculation, sexual, side effect

## Abstract

**Introduction:**

Venlafaxine is a serotonin-norepinephrine reuptake inhibitor and its extensive use for major depressive disorder and anxiety disorders. Although it has been reported that venlafaxine may have various side effects, as far as we know, spontaneous ejaculation(SE) has not been reported yet.

**Objectives:**

We aim to describe this clinical case with venlafaxine-induced SE and to discuss the possible etiological factors.

**Methods:**

Case report and literature review.

**Results:**

A 53-year-old male with generalized anxiety disorder was initiated venlafaxine treatment with 75 mg/day. After two months patient’s complaints partially regressed and the dose of venlafaxine treatment was increased to 150 mg/day. 10 days after the dose increase, the patient applied with the complaint of SE 2-3 times a day. No urological etiology was found. During outpatient follow-ups, after the 5 days from reducing the daily dose to 75 mg/day, SE complaint completely regressed. After following couple of months, the patient by himself, increased the dose of venlafaxine to 150 mg/day without consulting a psychiatrist. Then SE recurred approximately 15 days later. Venlafaxine treatment dose was reduced again to 75 mg/day and urological complaints spontaneously regressed.

**Conclusions:**

SE, a rare sexual side effect, represents ejaculation that occurs involuntarily and in the absence of any sexual stimuli. The possible mechanism of SE, detected as a side effect in our case, may be that increased adrenergic activity reduces ejaculatory latency and triggers spontaneous ejaculation. Antidepressant-associated sexual dysfunction could be a dose-dependent adverse event. Therefore, reducing the dosage of the treatment to a minimum effective dose could be an option.

**Disclosure:**

No significant relationships.

